# Comparable outcomes with anti-thymocyte globulins versus basiliximab in kidney transplantation from controlled circulatory death donors

**DOI:** 10.3389/ti.2026.15318

**Published:** 2026-06-01

**Authors:** Martin Deslais, Alice Koenig, Fanny Buron, Gabriel Ouellet, Maud Rabeyrin, Cécile Picard, Frédérique Dijoud, Renaud Snanoudj, Mohamad Zaidan, Florent von Tokarski, Arwa Jalal Eddine, Moglie Le Quintrec, Jean-Emmanuel Serre, Alice Corbel, Adrien Flahault, Magali Giral, Christophe Masset, Dany Anglicheau, Olivier Aubert, Antoine Sicard, Clément Gosset, Carmen Lefaucheur, Gillian Divard, Christophe Mariat, Quentin Monchal, Xavier Matillon, Anne-Claire Lukaszewicz, Valérie Dubois, Emmanuel Morelon, Olivier Thaunat, Xavier Charmetant

**Affiliations:** 1 Department of Transplantation, Nephrology and Clinical Immunology, Edouard Herriot Hospital, Hospices Civils de Lyon, Lyon, France; 2 Claude Bernard University (Lyon 1), Villeurbanne, France; 3 Department of Pathology, Groupement Hospitalier Est, Hospices Civils de Lyon, Bron, France; 4 Department of Nephrology, Dialysis, and Transplantation, Bicêtre Hospital, AP-HP, Le Kremlin-Bicêtre, France; 5 Department of Nephrology, Hemodialysis and Kidney Transplantation, Foch Hospital, Suresnes, France; 6 Department of Nephrology-Dialysis-Transplantation, University Hospital Centre of Montpellier, Montpellier, France; 7 Department of Nephrology-Dialysis-Transplantation, University Hospital Centre of Nancy, Nancy, France; 8 CHU Nantes, Nantes Université, Service de Néphrologie, Institut de Transplantation Uro-Néphrologique, Nantes, France; 9 Nantes Université, CHU Nantes, INSERM, Center for Research in Transplantation and Translational Immunology, UMR 1064, Nantes, France; 10 Department of Kidney and Metabolic Diseases, Transplantation and Clinical Immunology, Necker Hospital, Assistance Publique - Hôpitaux de Paris, Université Paris Cité, Paris, France; 11 Department of Nephrology-Dialysis-Transplantation, University Hospital Centre of Nice, Nice, France; 12 Department of Nephrology-Dialysis-Transplantation, University Hospital Centre of Saint Louis, Paris, France; 13 Nephrology, Dialysis and Renal Transplantation Department, Hôpital Nord, Centre Hospitalier Universitaire de Saint-Etienne, Jean Monnet University, Lyon, France; 14 Department of Urology and Transplant Surgery, Edouard Herriot Hospital, Hospices Civils de Lyon, Lyon, France; 15 Anesthesia and Critical Care Medicine Department, Edouard Herriot Hospital, Hospices Civils de Lyon, Lyon, France; 16 HLA Laboratory, French National Blood Service (EFS), Décines, France

**Keywords:** antithymocyte globulin, basiliximab, DCD (donation after circulatory death), delayed graft function, induction

## Abstract

Due to organ shortage, donation after circulatory death (DCD) has increased. As DCD kidney grafts have higher rates of delayed graft function (DGF), the French national protocol mandates the use of hypothermic machine perfusion and routine anti-thymocyte globulin (ATG) induction therapy. However, evidence favoring ATG over interleukin-2 receptor antagonists, such as basiliximab, remains scarce. We retrospectively analysed a single-center cohort of 158 low immunological risk patients who underwent DCD kidney transplantation between 2015 and 2023. Patients transplanted before March 2020 received ATG (n = 64), while those transplanted thereafter (during and after the COVID-19 pandemic) received basiliximab (n = 94). Baseline characteristics were comparable, except for recipient age (lower in the basiliximab group), and cold ischemia times were similar. There were no differences in primary non-function, DGF rates (17% after ATG versus 12% after basiliximab), biopsy-proven rejection, one-year renal function or graft survival. ATG was associated with prolonged EBV and BK viremia without increased complications. Hospital stays were similar, but ATG induction was 1.7 times more expensive. These findings were confirmed in an independent French multicenter cohort (n = 506). In conclusion, basiliximab appears to be a cost-effective alternative to ATG for low-immunological-risk patients receiving DCD kidney grafts, for whom ATG is not clearly indicated.

## Graphical Abstract

**Figure:**
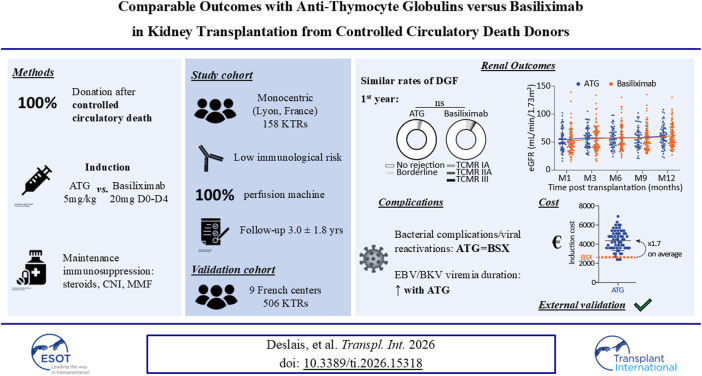

## Introduction

Kidney transplantation is the preferred treatment for patients with end-stage renal disease (ESRD), offering both improved quality of life [1] and significantly better survival [2, 3] for only a fraction of the costs of hemodialysis. However, due to the shortage of organ available there is a growing imbalance between the number of patients waiting for a graft and the number of transplantations performed each year [4]. As a result, many patients die each year while waiting for a compatible graft [4, 5].

To address the problem of organ shortage and optimize access to transplants, various strategies have been implemented to expand the donor pool. These include regenerative medicine approaches and xenotransplantation - currently still in the research phase [6, 7] - as well as the already established use of living donors and the extension of criteria for transplantation from deceased donors. In France, the “Maastricht 3 program”, launched in 2015, enabled the procurement of organs from donors after controlled circulatory death (DCD) [8]. This program has significantly contributed to increase the availability of kidney transplants. Between 2014 and 2024, the total number of kidney transplants from deceased donors in France rose by 16.2% (2718 in 2014 vs. 3159 in 2024), with DCD donors accounting for nearly all this increase, while the number of donations after brain dead remained stable [4]. A similar trend has been observed in the United States [5].

However, due to the unavoidable warm ischemia time before organ retrieval, DCD transplants are inherently associated with an increased risk of delayed graft function (DGF) [9, 10]. In France, several measures have been proposed to mitigate the risk of DGF, and, more critically, primary non-function (PNF). First, all grafts from DCD donors must undergo hypothermic machine perfusion, which has been shown to reduce the risk of DGF [11, 12] particularly in donors aged over 60 [13]. Second, cold ischemia time (CIT), which is a known variable associated with DGF [12–14], is limited to 18 h (with a strong recommendation to keep it below 12 h). Third, at the time the program was set up, the French regulatory agency recommended the systematic use of depleting induction therapy with polyclonal antibodies (either anti-thymocyte globulins (ATG) or anti-lymphocyte globulins) to mitigate ischemia-reperfusion injury [15] and to promote early graft function [16] by allowing delayed introduction of calcineurin inhibitors. Interestingly, the combination of hypothermic machine perfusion and polyclonal antibodies may have synergistic effects in reducing graft immunogenicity and lowering rejection rates following transplantation with a DCD graft. Hypothermic perfusion facilitates the release of tissue-resident leukocytes into the perfusion fluid [17], while polyclonal antibodies deplete circulating passenger leukocytes. Given that several allorecognition pathways rely directly on donor passenger leukocytes [18–20], this approach could contribute to a longer-term reduction in rejection.

Notably, the expected short and long-term efficacy of polyclonal antibodies after DCD transplantation has never been prospectively compared with interleukin-2 receptor antagonists (IL-2RA) such as basiliximab or daclizumab. Therefore, we retrospectively analyzed prospectively collected data from a single center to compare the short-, medium-, and long-term outcomes of ATG versus basiliximab induction in kidney transplant recipients from DCD donors and assess their cost-effectiveness. These findings were subsequently evaluated in an independent validation cohort.

## Patients and methods

### Study design

The study cohort consisted in a monocentric cohort of kidney transplant patients from Lyon University Hospital, France. It was used to retrospectively compare the impact of induction treatment in patients receiving kidneys from controlled circulatory death donors. All patients receiving a kidney transplantation from a DCD donor between January 1, 2015 and December 31, 2023 were enrolled. Data were prospectively collected. The validation cohort (DIVAT, Données Informatisées et VAlidées en Transplantation) included 506 adult recipients of DCD kidney transplants from 9 French centers [Necker (Paris), Saint Louis (Paris), Foch (Suresnes), le Kremlin-Bicêtre, Saint-Etienne, Nantes, Nancy, Montpellier, Nice] between January 1, 2015, and December 31, 2023.

### Inclusion and exclusion criteria

In France, the Maastricht III program, which defines the rules for transplants using controlled DCD donors, imposes strict conditions. Donors must be strictly under the age of 70 and must not have acute organ failure. The agonic phase must last less than 3 h and the functional warm ischemia phase (time interval during which the organs are hypoperfused and then no longer perfused at all due to circulatory failure) less than 120 min for the kidneys. *Ex vivo* hypothermic perfusion is mandatory and must last at least 2 hours, while CIT must be < 18h. Recipients can only receive a first, HLA-compatible transplant.

Patients who received both available induction treatments were excluded from the analysis.

### Definition of delayed graft function

Delayed graft function was defined as the need for at least one hemodialysis session after the transplantation.

### Histological evaluation

Light microscopy was performed on systematic or “for cause” kidney biopsy samples fixed in formalin-acetic alcohol after staining with haematoxylin and eosin. Histological lesions were scored according to the Banff 2022 classification [21].

### Statistical analysis

Qualitative variables were expressed as percentages and compared with the chi-square test or Fisher’s exact test when the conditions of application of chi-square were not met. Quantitative variables were expressed as mean ± SD and compared using Student’s t-test. All tests were two-sided. When repeated measurements of the same parameter were performed over time, two-way ANOVA or mixed effects model were used with Sidak’s multiple comparisons test. No imputation was performed. Analyses were performed using variable-specific complete-case analyses, excluding only observations with missing data for the variable under consideration. Incidence and survival data were analysed by Kaplan-Meier curve and compared using a log-rank test. A multivariable Cox proportional hazards model was used to assess patient and graft survival in the validation cohort. All data were analysed using Prism (GraphPad) or the R software. All analyses were performed using R Statistical Software (v4.5.1); R Core Team [22].

### Study approval

The study was carried out in accordance with French legislation on biomedical research and the Declaration of Helsinki. All patients gave informed consent for the utilization of clinical data [Données Informatiques Validées en Transplantation (DIVAT cohort)] for research purpose (www.divat.fr, CNIL (Commission Nationale de l’Informatique et des Libertés) no. 914184, ClinicalTrials. gov: NCT02900040).

## Results

### Impact of the COVID-19 pandemic on induction therapy selection for DCD kidney transplants

**FIGURE 1:**
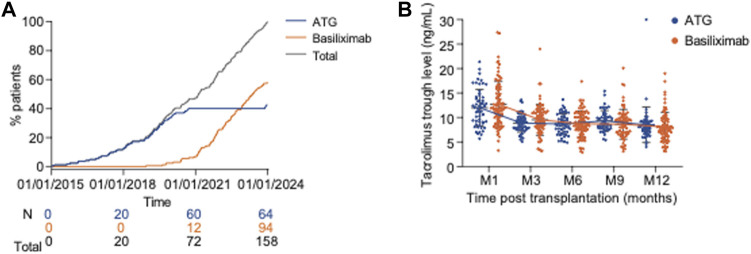
Evolution of Immunosuppressive Strategies Over Time. **(A)** Curve depicting the incidence of transplanted patients receiving a graft from a DCD donor, overall (black curve), induced with ATG (blue curve), or induced with basiliximab (orange curve). **(B)** Evolution of tacrolimus levels over time based on the induction therapy received. Mixed effect model, ns. M, month.

Between January 2015 and December 2023, 163 patients received a transplant from a DCD donor. All received induction treatment with ATG (Thymoglobulin, Sanofi ®, target dose 5 mg/kg) or an IL-2RA, basiliximab (Simulect, Novartis ®, 20 mg day 0 and day 4). As they did not tolerate ATG, 5 patients were switched to basiliximab and received both molecules; they were therefore excluded from the study. Of the remaining 158 patients, the vast majority of those transplanted before the onset of COVID-19 pandemic in France (March 2020) have received ATG as an induction, as specified in the National protocol [23]. To limit the risk of COVID-19 induced mortality in the post-transplantation period [24, 25], almost all patients transplanted after March 2020 were induced with basiliximab (Figure 1A). However, the change in induction treatment was not accompanied by changes in the maintenance immunosuppressive regimen. In particular, the target residual doses of calcineurin inhibitors were the same for all patients (Figure 1B; of note, the few patients (n = 5; 3%) on ciclosporin were excluded from this analysis), regardless of the induction treatment.

Taking advantage of this situation, we underwent the present study aiming at comparing the effect of induction on the prognosis of transplants with DCD donors. Therefore, transplant outcomes (in terms of renal parameters and complications) were compared between the 64 patients induced with ATG and the 94 patients induced with basiliximab, whose characteristics are presented in Table 1. Briefly, the mean recipient age was 56.2 ± 11.7 and 52.0 ± 12.6 years and the mean donor age was 50.6 ± 12.6 and 50.8 ± 13.6 years, in ATG and basiliximab groups, respectively. The type of initial kidney disease leading to end-stage renal disease was similar in both groups. The mean follow-up was 3.0 ± 1.8 years; nine patients died during follow-up.

**TABLE 1:** Patients characteristics–Original cohort.

Mean ± SD or n (%)	Whole cohort N = 158	ATG N = 64	BSX N = 94	p-value
Recipient characteristics at the time of transplantation				
Female	47 (30)	23 (36)	24 (26)	0.160
Age (years)	54 ± 12.4	56 ± 11.7	52 ± 12.6	0.046
BMI (kg/m^2^)	26.2 ± 4.6	26.3 ± 5.1	26.1 ± 4.3	0.791
Blood group	​	​	​	0.044
A	64 (41)	20 (31)	44 (47)	
B	20 (13)	13 (20)	7 (8)	
O	65 (41)	26 (41)	39 (41)	
AB	9 (5)	5 (8)	4 (4)	
Renal disease	​	​	​	0.539
Diabete mellitus	24 (15)	10 (16)	14 (15)	
Glomerulonephritis	43 (27)	17 (27)	26 (28)	
Tubular-interstitial, genetic	12 (8)	20 (31)	19 (20)	
Vascular	22 (14)	11 (17)	11 (12)	
Uropathy	1 (1)	0	1 (1)	
Undetermined	34 (21)	11 (17)	23 (24)	
Anti-HLA antibody DSA	31 (20) 0	13 (20) 0	18 (19) 0	0.857 NA
Donor characteristics				
Female	43 (27)	15 (23)	28 (30)	0.379
Age (years)	51 ± 13.2	51 ± 12.6	51 ± 13.6	>0.999
BMI (kg/m^2^)	25.3 ± 5.3	25.5 ± 5.3	25.1 ± 5.3	0.642
Transplantation characteristics				
Cold ischemia time (min)	776 ± 244	787 ± 214	769 ± 264	0.651
Perfusion machine	158 (100)	64 (100)	94 (100)	NA
No. of HLA A/B/DR mismatches	3.7 ± 1.1	3.7 ± 1.1	3.7 ± 1.2	>0.999
Induction therapy dose (mg/kg for ATG, mg for BSX)	ND	4.9 ± 0.6	40 ± 2	NA
Maintenance immunosuppression at hospital discharge	​	​	​	​
Tacrolimus	152 (96)	58 (91)	94 (100)	0.009
Cyclosporine	5 (3)	5 (8)	0	​
MMF	154 (97)	63 (98)*	91 (97)	0.647
Steroids mTORi	157 (99)*	63 (98)*	94 (100)	0.405
Belatacept	2 (1)	0	2 (2)	0.515
​	1 (1)	0	1 (1)	>0.999
CMV status	​	​	​	0.200
D^−^/R^-^	29 (18)	14 (22)	15 (16)	
D^+^/R^-^	21 (13)	5 (8)	16 (16)	
R^+^	108 (69)	45 (70)	63 (68)	
EBV status	​	​	​	0.247
D^−^/R^-^	1 (1)	0	1 (1)	
D^+^/R^-^	3 (2)	0	3 (3)	
R^+^	154 (97)	64 (100)	90 (96)	

Abbreviations: ATG, anti-thymocyte globulins; BSX, basiliximab; BMI, body mass index; DSA, donor-specific antibodies; ND, not determined; MMF, mycophenolate mofetil; mTORi, mechanistic target of rapamycin inhibitor; CMV, cytomegalovirus; EBV, Epstein-Barr virus; D/R, donor/recipient; *, death of a patient at day 17 of transplantation.

### The type of induction therapy has no impact on renal graft function or immunological outcomes

**FIGURE 2:**
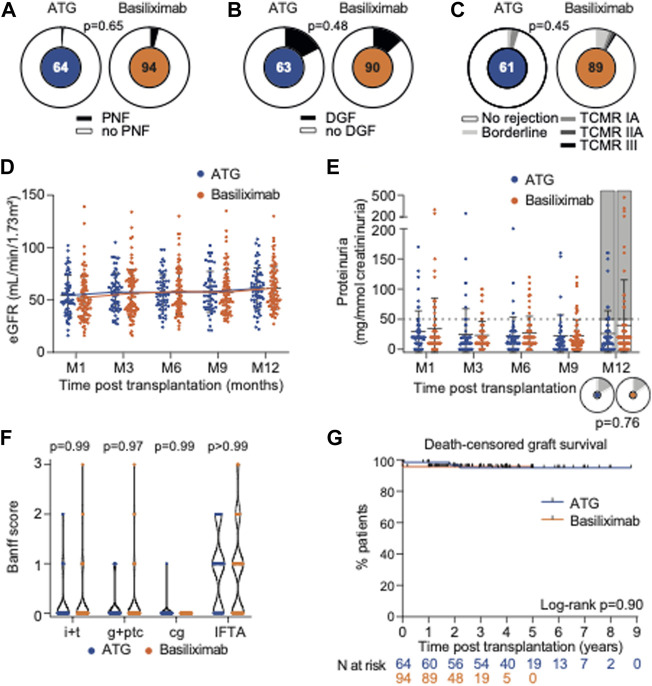
Comparison of renal functional and immunological outcomes in patients receiving ATG or basiliximab. **(A–C)** The prevalence of **(A)** primary non-function (PNF), **(B)** delayed graft function (DGF) and **(C)** rejection was compared between groups **(A)** in the entire cohort, **(B)** in patients without PNF and **(C)** in patients without PNF who were alive at the one-year follow-up. Fisher’s test **(A,B)** and chi-square test **(C)**, ns. **(D,E)** Evolution of **(D)** estimated glomerular filtration rate (eGFR) and **(E)** proteinuria levels over time based on the induction therapy received. Mixed effect models, ns. **(F)** Comparison of the Banff i + t, g + ptc, cg and IF/TA scores between groups in the one-year biopsy. Two-way ANOVA, ns. **(G)** Kaplan-Meier survival curve for death-censored graft survival according to induction therapy. M; month. TCMR; T-cell mediated rejection.

The cold-ischemia time was comparable between the groups (787 ± 214 min in ATG group vs. 769 ± 264 min in basiliximab group; unpaired t-test, p = 0.66; Table 1), and all kidneys were preserved on a perfusion machine. The rate of PNF was low (5; 3.16%) and did not differ according to the induction received (1 [1.56%] patient in ATG group, vs. 4 [4.25%] patients in basiliximab group; Fisher’s exact test, p = 0.65; Figure 2A). Among the remaining 153 patients, 15% experienced DGF, 11 in ATG group and 12 in basiliximab group (chi-square test, p = 0.48; Figure 2B). Thus, induction does not affect the early outcomes after transplantation.

Since some studies have indicated a lower risk of biopsy-proven rejection with ATG induction compared to IL-2RA [26], we sought to determine whether this also holds true for DCD transplants, considered more immunogenic [27]. In our cohort, patients had very low levels of immunization (19.6% with preformed anti-HLA antibodies, none with preformed donor-specific antibodies; chi-square test for comparison between groups, p = 0.86; Table 1), and the number of HLA-A/B/DR mismatches was also similar between the groups (3.7 ± 1.1 and 3.7 ± 1.2 in ATG and basiliximab groups, respectively; chi-square test, p = 0.86; Table 1). Overall, 5 patients experienced full blown T-cell mediated rejection (2 and 1 grade IA, 0 and 1 grade IIA, 0 and 1 grade III, in ATG and basiliximab groups, respectively) during the first year of follow-up, whereas one patient with concomitant BK nephropathy was excluded from this analysis. Over the same period of 1 year of follow-up, 6 other patients had borderline lesions (1 in ATG group and 5 in basiliximab group). The intensity of inflammatory lesions (borderline lesions or TCMR) did not differ between the groups (chi-square test, p = 0.45; Figure 2C). Of these 11 patients, all but two (with borderline lesions) were treated with corticosteroids and the control biopsy performed at 192 ± 83 days showed resolution of inflammation in all cases. Two of these 9 treated patients developed post-transplant diabetes mellitus. In addition, three patients in the basiliximab group developed microvascular inflammation (MVI) without DSA.

The evolution of renal function and proteinuria during the first year was comparable regardless of induction (Figures 2D,E). Patients had a mean one-year estimated glomerular filtration rate of 61.0 ± 19.6 mL/min/1.73 m^2^ and 61.3 ± 20.3 mL/min/1.73 m^2^ in ATG and basiliximab groups, respectively (unpaired t-test, p = 0.93; Figure 2D). At 1 year, among patients with an available proteinuria measurement, 10 of 59 and 12 of 80 had significant proteinuria ≥50 mg/mmol creatininuria in ATG and basiliximab groups, respectively (chi-square test, p = 0.76; Figure 2E).

At the systematic one-year evaluation, 102 patients had a biopsy (41 in the ATG group and 61in the basiliximab group, mean delay 383 ± 23 days post transplantation [range 275–455 days]). Among all biopsies for which the following Banff scores were available, there was no difference in terms of tubulointerstitial (i + t; evaluated on 91 biopsies) or microvascular (g + ptc; 89 biopsies) acute inflammatory lesions, nor in chronic glomerular lesions (cg; 90 biopsies) or interstitial fibrosis/tubular atrophy (IFTA; 88 biopsies; Figure 2F). Very few patients developed *de novo* donor-specific antibodies (2 in ATG group, 1 in basiliximab group, log-rank test, p = 0.36).

Finally, to integrate all the risks (PNF, DGF, rejection…) that could potentially affect graft function on the long term, we compared the death-censored graft survival of the two groups and found no difference (log-rank test, p = 0.90; Figure 2G).

### The type of induction therapy has no impact on infectious and cancer complications

Having demonstrated that graft survival was similar in the two groups of patients, we wanted to compare the safety of the two induction therapies.

**FIGURE 3:**
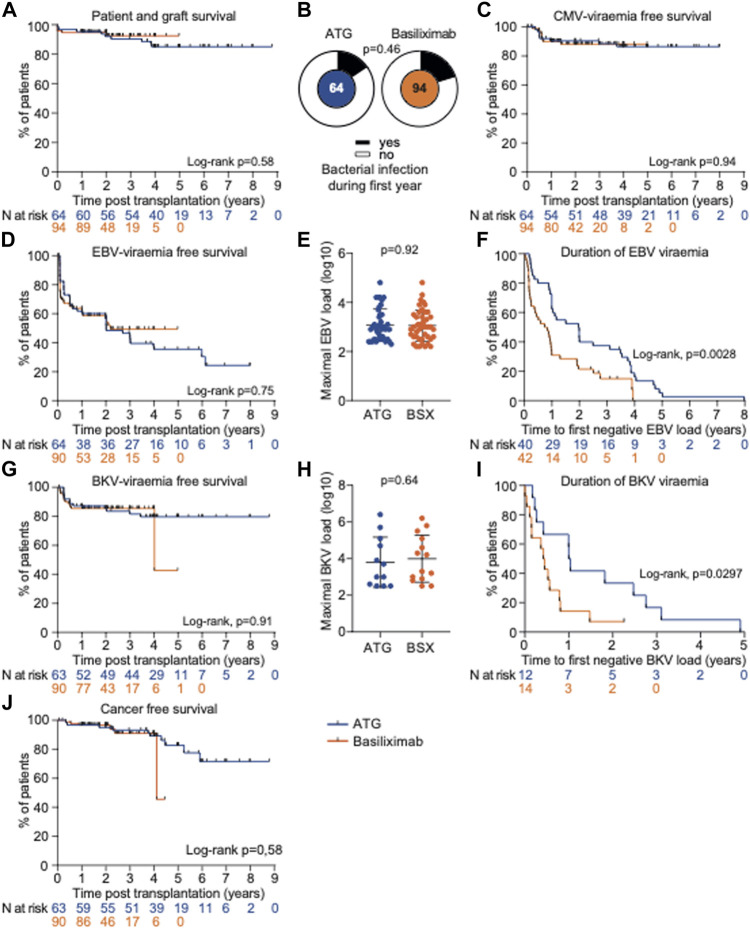
Comparison of infectious and cancer complications in patients receiving ATG or basiliximab. **(A)** Kaplan-Meier survival curve for patient and graft survival according to induction therapy. **(B)** The prevalence of bacterial infections during the first year was compared between each group in the entire cohort. Chi-square test, ns. **(C,D)** Kaplan-Meier survival curve for **(C)** CMV-viraemia and **(D)** EBV-viraemia free survival according to induction therapy in the entire cohort. **(E)** Comparison of the maximal EBV viral load between groups. Student’s t-test, ns. **(F)** Kaplan-Meier curve of the delay between EBV reactivation and viral clearance according to induction therapy. **(G)** Kaplan-Meier survival curve for BKV-viraemia free survival according to induction therapy in patients without primary non function. **(H)** Comparison of the maximal BKV viral load between groups. Student’s t-test, ns. **(I)** Kaplan-Meier curve of the delay between the first BKV viraemia and viral clearance according to induction therapy. **(J)** Kaplan-Meier survival curve for cancer free survival according to induction therapy.

Patient and graft survival were similar between the 2 groups (log-rank test, p = 0.58; Figure 3A). The number of bacterial complications (including bacteremia, pyelonephritis and bacterial pneumonia) during the first year was equivalent (chi-square test, p = 0.46; Figure 3B). Regarding viral complications, we observed no difference in the incidence of cytomegalovirus (CMV) viremia between the two groups (log-rank test, p = 0.94; Figure 3C). However, this result should be interpreted with caution because patients received protocolized prophylaxis against CMV with valganciclovir, the duration of which varied according to the serological status of the donor/recipient pair (6 months for the high-risk seropositive donor/seronegative recipient group [D+/R-] and 3 months for intermediate-risk group [R+]). Although the difference was not statistically significant, there were more D+/R- high-risk pairs in the basiliximab than in the ATG group (16% vs. 8% respectively; Table 1). Regarding EBV, there were only 4 high-risk pairs (D+/R-) and all received basiliximab as induction. In the rest of patients (D+/R+ or D-/R+), the incidence of EBV reactivation was comparable between groups (log-rank test, p = 0.75; Figure 3D) and the maximum viral load was similar (mean maximum viral load 3.1 ± 0.7 log vs. 3.1 ± 0.6 log in the ATG and basiliximab groups, respectively; unpaired t-test, p = 0.92; Figure 3E), but the duration of viremia was significantly longer in patients who had received ATG (log-rank test, p = 0.0028; Figure 3F). Despite this difference, no patient of the cohort developed EBV-related post-transplant lymphoproliferative disease during the follow-up period. For the analysis focusing on BK virus, we excluded from the analysis the 5 patients who experienced PNF and remained on dialysis. The results were reminiscent to those obtained for EBV. The incidence of viremia (log-rank test, p = 0.91; Figure 3G), and maximum viral loads (mean maximum viral load 3.8 ± 1.4 log vs. 4.0 ± 1.3 log in ATG and basiliximab groups, respectively; unpaired t-test, p = 0.71; Figure 3H), were similar in the two groups but patients from the ATG group exhibited longer duration of viremia (log-rank test, p = 0.0297; Figure 3I). However, this difference did not translate into a higher risk of BK virus-associated nephropathy (BKVAN), as only 4 patients developed BKVAN, 1 in the group ATG and 3 in the group basiliximab (log-rank test, p = 0.41).

Finally, the risk of cancer was equivalent in each group (log-rank test, p = 0.58; Figure 3J).

### Validation of these results in an independent cohort

To assess the external validity of our findings, we analysed an independent validation cohort comprising 521 kidney transplant recipients from nine additional French centers who received a DCD graft between January 2015 and December 2023. Fifteen patients were excluded due to missing induction data. Among the 506 patients analysed, 408 received ATG and 98 received basiliximab. The characteristics of these patients are presented in Table 2. Of note, a higher proportion of patients in this cohort were immunised, with 61% having preformed anti-HLA antibodies and 15% having day-0 DSA. Furthermore, 7% of grafts in both groups were not preserved using a perfusion machine.

**TABLE 2:** Patients characteristics–Validation cohort.

Mean + SD or n (%)	Whole cohort N = 506	ATG N = 408	BSX N = 98	p-value
Recipient characteristics at the time of transplantation				
Female	176 (35)	150 (37)	26 (27)	0.073
Age (years)	54 ± 12.6	54 ± 12.6	52 ± 12.6	0.198
BMI (kg/m^2^)	25.3 ± 4.4^1^	25.4 ± 4.4^1^	24.5 ± 4.4	0.049
Blood group	​	​	​	0.282
A B O AB ND	218 (43) 46 (9) 216 (43) 21 (4) 5 (1)	171 (42) 39 (10) 181 (44) 15 (3) 2 (1)	47 (48) 7 (7) 35 (36) 6 (6) 3 (3)	
Renal disease	​	​	​	0.888
Diabete mellitus Glomerulonephritis Tubular-interstitial, genetic Vascular Undetermined	52 (10) 120 (24) 196 (39) 64 (13) 74 (14)	41 (10) 97 (24) 155 (38) 54 (13) 61 (15)	11 (11) 23 (24) 41 (42) 10 (10) 13 (13)	
Anti-HLA antibody DSA	266 (61^2^) 63 (15^5^)	200 (60^3^) 47 (14^6^)	66 (68^4^) 16 (16^7^)	0.203 1
Donor characteristics				
Female	144 (28)	121 (30)	23 (23)	0.274
Age (years)	52 ± 12.9^8^	52 ± 12.9	52 ± 13.4^8^	0.959
BMI (kg/m^2^)	26.0 ± 5.5^9^	26.0 ± 5.6^9^	25.9 ± 5.5	0.846
Transplantation characteristics				
Cold ischemia time (min)	614 ± 247^10^	607 ± 250^11^	643 ± 234^12^	0.181
Perfusion machine	450 (93^13^)	373 (93^14^)	77 (92^15^)	0.580
No. of HLA A/B/DR mismatches	3.5 ± 1.1^16^	3.5 ± 1.1	3.5 ± 1.1^16^	0.879
Maintenance immunosuppression at hospital discharge	​	​	​	​
Tacrolimus Cyclosporine MMF Steroids mTORi Belatacept	441 (87) 63 (12) 485 (96) 497 (98) 17 (3) 0	343 (84) 63 (15) 401 (98) 405 (99) 2 (1) 0	98 (100) 0 84 (86) 92 (94) 15 (15) 0	<0.001 <0.001 0.002 <0.001
CMV status	​	​	​	0.016
D^−^/R^-^ D^+^/R^-^ R^+^ ND	124 (24) 86 (17) 292 (58) 4 (1)	94 (23) 63 (15) 248 (61) 3 (1)	30 (31) 23 (23) 44 (45) 1 (1)	
EBV status	​	​	​	<0.001
D^−^/R^-^ D^+^/R^-^ R^+^ ND	1 (0) 30 (6) 472 (93) 3 (1)	1 (0) 16 (4) 389 (95) 2 (1)	0 14 (14) 83 (85) 1 (1)	

Abbreviations: ATG, anti-thymocyte globulins; BSX, basiliximab; BMI, body mass index; DSA, donor-specific antibodies; ND, not determined; MMF, mycophenolate mofetil; mTORi, mechanistic target of rapamycin inhibitor; CMV, cytomegalovirus; EBV, Epstein-Barr virus; D/R, donor/recipient.

1 ND for 1 recipients.

2 ND for 73 recipients.

3 ND for 72 recipients.

4 ND for 1 recipient.

5 ND for 83 recipients.

6 ND for 82 recipients.

7 ND for 1 recipient.

8 ND for 1 donor.

9 ND for 2 donors.

10 ND for 6 grafts.

11 ND for 4 grafts.

12 ND for 2 grafts.

13 ND for 23 grafts.

14 ND for 9 grafts.

15 ND for 14 grafts.

16 ND for 1 D/R pair.

**FIGURE 4:**
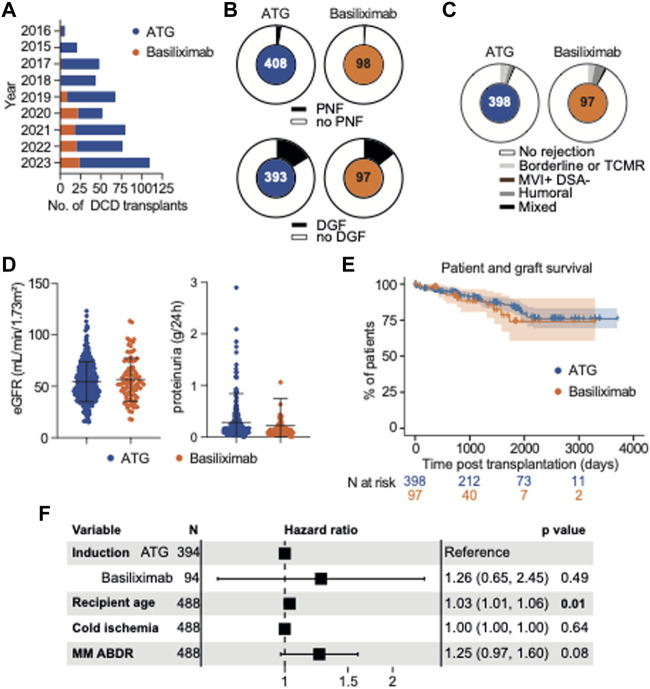
Renal functional and immunological outcomes in the validation cohort. **(A)** Histogram showing the annual number of DCD transplants by induction therapy in the validation cohort. **(B)** Prevalence of primary non-function (PNF) in the entire validation cohort and delayed graft function (DGF) in patients without PNF. Chi-square and Fisher’s exact test, ns. **(C)** Prevalence of rejection in patients without PNF during first year of follow-up. Chi-square test, ns. **(D)** Comparison of estimated glomerular filtration rate (eGFR) and proteinuria level at 1 year’s evaluation. Student’s t-test, ns. **(E)** Kaplan-Meier survival curve for death-censored graft survival according to induction therapy. **(F)** Forest plot representing the results of the multivariable Cox regression analysis. Abbreviations: MVI, microvascular inflammation; DSA, donor-specific antibodies.

The distribution of induction regimens was more balanced over time than in the study cohort (Figure 4A). The rates of PNF, DGF (after exclusion of PNF), and rejections occurring during the first-year post-transplantation were similar between groups (Figures 4B,C). Kidney function and proteinuria at 1 year were also comparable between patients receiving ATG or basiliximab (Figure 4D).

Protocol biopsies were less systematically performed in this cohort, with fewer than one third of patients undergoing a one-year biopsy; therefore, no biopsy analysis at 1 year is reported.

Importantly, death-censored graft survival did not differ between the groups (log-rank test, p = 0.44; Figure 4E). Multivariable Cox regression adjusted for recipient age, HLA-A/B/DR mismatches and cold ischemia time confirmed that survival was comparable between basiliximab and ATG groups (HR 1.26, 95% CI 0.65–2.45; p = 0.49; Figure 4F).

**FIGURE 5:**
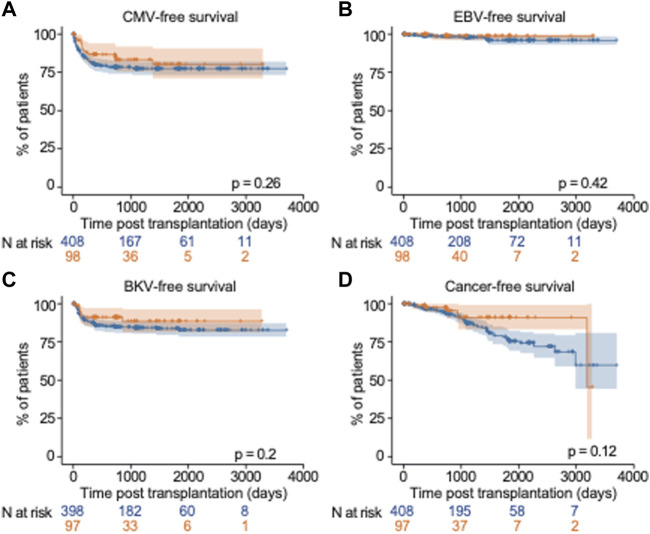
Viral and cancer complications in the validation cohort. **(A,B)** Kaplan-Meier survival curve for **(A)** CMV-viraemia and **(B)** EBV-viraemia survival according to induction therapy in the entire cohort. **(C)** Kaplan-Meier survival curve for BKV-viraemia free survival according to induction therapy in patients without primary non function. **(D)** Kaplan-Meier survival curve for cancer free survival according to induction therapy.

From a safety perspective, the two groups were compared in terms of viral and malignancy-related complications. The incidences of CMV, EBV and BKV viremia were comparable (log-rank tests, p = 0.26, p = 0.42 and p = 0.2, respectively; Figures 5A–C). A non-significant trend towards an increased risk of cancer (all types combined) was observed in the ATG group (log-rank test, p = 0.12; Figure 5D).

### ATG vs. basiliximab induction: comparable efficacy and risks, divergent costs

**FIGURE 6:**
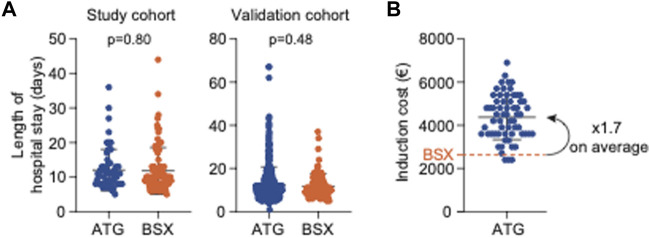
Comparison of hospital stay duration in patients receiving ATG or basiliximab and treatment costs. **(A)** Comparison of the length of hospital stay between groups in the study (left) and the validation (right) cohorts. Student’s t-test, ns. **(B)** Comparison of the costs of each treatment. The dashed line represents the fixed cost of induction with basiliximab.

Basiliximab-treated patients exhibited slightly more early inflammatory kidney lesions without long-term graft impact, while ATG-treated patients experienced prolonged EBV and BKV viremia without apparent severity. These non-significant differences balance the risks, offering no clear advantage for either agent. A pragmatic approach is to consider tolerance - measured here by the length of the initial hospital stay - and cost. Hospital stays were similar in both groups (mean 12 ± 6 days for ATG vs. 12 ± 7 days for basiliximab, p = 0.80; and 12 ± 8 days for ATG vs. 12 ± 7 days for basiliximab, p = 0.48; in the study and validation cohorts, respectively; Figure 6A). However, at our institution, ATG induction is, on average, 1.7 times (range 0.92–2.65) more expensive than basiliximab (€300 per ATG vial, median 15 vials per patient versus €1,315 per basiliximab vial, 2 vials per patient; Figure 6B). Therefore, we conclude that in the absence of a compelling clinical reason to prefer ATG, basiliximab appears to be a reasonable and cost-effective choice for induction in DCD kidney transplant recipients.

## Discussion

In this study, we demonstrate, in two independent cohorts including a total of 664 patients, that induction therapy with ATG or basiliximab is associated with similar outcomes following kidney transplantation from DCD donors in recipients with a low immunological risk. ATG did not provide any advantage in terms of DGF or rejection incidence. However, it was also not associated with a higher risk of infectious or cancer-related complications.

In fact, at the time the program was set up in France, the rationale for preferring ATG to basiliximab appeared to be weak, as highlighted by the British and European recommendations on this topic [28, 29]. Since then, two studies have specifically compared ATG and IL-2RA in DCD kidney transplantation. In the first study [30], IL2-RA induction was not associated with a higher risk of DGF. In the second study [31], ATG was associated with a lower incidence of acute rejection but did not reduce the risk of DGF. Notably, the rates of DGF in both studies were very high, compared to our study which found around 15% DGF, with comparable rates between the groups. Limiting ischemia as much as possible seems to be crucial for these particular grafts, but our CIT were comparable to those previously published. Therefore, this difference is more likely due to the systematic use of normothermic regional perfusion [32] and hypothermic perfusion machine [11, 12], which was not the case in the two published studies. Consequently, our results on induction must be interpreted as part of a strict protocol, which imposes the two technical constraints mentioned above. They cannot therefore be extrapolated outside this specific framework.

One of the strengths of our study is that many patients of the cohort study (65.8% of those with a functioning graft at 1 year) had a screening renal biopsy 1 year post-transplantation, allowing for the detection of subclinical graft inflammation that would not have been seen based only on for cause biopsy and functional parameters [33]. Additionally, the relatively long follow-up period reinforces the robustness of our conclusions.

The theoretical benefit of ATG in mitigating ischemia-reperfusion injury is based on experimental data suggesting that these polyclonal cocktails also contain antibodies specific of adhesion molecules [34, 35] potentially limiting ischemia-reperfusion damage [15] by modulating the inflammatory infiltrate of the graft. As ischemia-reperfusion injury can theoretically still contribute to long-term graft remodeling, ATG should limit chronic graft dysfunction (which can be evaluated by cg and IF/TA lesions). However, our findings do not support any benefit of ATG, or this mechanism may not be relevant in DCD transplant recipients.

Interestingly, we also observed lower rates of T cell-mediated rejection in the basiliximab group compared to previous reports, despite no apparent differences in maintenance immunosuppression. One possible explanation is that perfusion may help clear ischemia-induced pro-inflammatory mediators and donor-derived immune cells, thereby reducing graft immunogenicity and potentially allowing for less intensive immunosuppression. These results confirm that ATG and IL-2RA give similar results in terms of rejection in patients at low immunological risk, as shown in previously published studies in kidney transplantation with brain-dead donors [36–38].

While we expected more infectious complications, particularly viral, in the ATG group [39–44], we did not observe any excess risk associated with the use of this induction therapy in our study. This result has probably various explanations, such as the lower dose of ATG used in our study (5 mg/kg versus 5–10 mg/kg in other studies), and the systematic use of anti-CMV prophylaxis in patients at risk.

Everything else being equal, it makes sense to prioritize a less expensive drug that does not require the use of animal for its production. While this study was not designed to assess environmental impact, it is reasonable to assume that monoclonal antibodies have a lower ecological footprint. Since one of the key advantages of transplantation over hemodialysis is its lower societal cost—both financially and in terms of resource consumption (such as plastic disposables and other materials)—optimizing certain parameters, such as induction, could further strengthen this economic and environmental benefit.

Our study has some limitations. First, while the cohort sizes are sufficiently large to support statistical conclusions, the population is highly homogeneous, which limits the generalizability of our findings to more diverse groups, such as older recipients or those at higher immunological risk. Second, the study is retrospective. However, as the choice of induction therapy in the study cohort was primarily determined by the transplantation period, clinician-driven selection bias was minimized. Notably, induction strategy was the only variable that differed between the two study periods. Furthermore, in the independent validation cohort, the use of induction therapy was more evenly distributed over time, with comparable results, suggesting that the potential bias mentioned above is likely negligible. Third, the follow-up duration may not have been sufficient to fully assess the long-term impact of induction therapy on late complications, such as cancer. Finally, although our study suggests a longer duration of BKV and EBV replication in the ATG group, these findings should be interpreted with caution due to the lack of standardized viral monitoring, which may have introduced bias into the results.

In conclusion, our study does not support selecting induction therapy based solely on DCD donor criteria, as it does not influence transplant outcomes. From a cost-effectiveness perspective, basiliximab should be the preferred choice for patients who do not require ATG to prevent the recurrence of their initial nephropathy. Further studies are needed to determine the optimal approach for high immunological risk recipients.

## Data Availability

The original contributions presented in the study are included in the article/supplementary material, further inquiries can be directed to the corresponding author.

## References

[1] Maglakelidze N Pantsulaia T Tchokhonelidze I Managadze L Chkhotua A . Assessment of health-related quality of life in renal transplant recipients and dialysis patients. Transplant Proc (2011) 43(1):376–9. 10.1016/j.transproceed.2010.12.015 21335226

[2] Tonelli M Wiebe N Knoll G Bello A Browne S Jadhav D Systematic review: Kidney transplantation compared with dialysis in clinically relevant outcomes. Am J Transplant (2011) 11(10):2093–109. 10.1111/j.1600-6143.2011.03686.x 21883901

[3] Wolfe RA Ashby VB Milford EL Ojo AO Ettenger RE Agodoa LYC Comparison of mortality in all patients on dialysis, patients on dialysis awaiting transplantation, and recipients of a first cadaveric transplant. N Engl J Med (1999) 341(23):1725–30. 10.1056/NEJM199912023412303 10580071

[4] Agence de la biomédecine. Agence de la biomédecine. Available online at: https://rams.agence-biomedecine.fr/greffe-renale-0 (Accessed April 9, 2025).

[5] Kidney - Scientific Registry of Transplant Recipients. Kidney - Scientific registry of transplant recipients. Available online at: https://srtr.transplant.hrsa.gov/ADR/Chapter?name=Kidney&year=2023 (Accessed April 9, 2025).

[6] Montgomery RA Stern JM Lonze BE Tatapudi VS Mangiola M Wu M Results of two cases of pig-to-human kidney xenotransplantation. N Engl J Med (2022) 386(20):1889–98. 10.1056/NEJMoa2120238 35584156

[7] Kawai T Williams WW Elias N Fishman JA Crisalli K Longchamp A Xenotransplantation of a porcine kidney for end-stage kidney disease. N Engl J Med (2025) 392(19):1933–40. 10.1056/NEJMoa2412747 39927618

[8] Lamy FX Atinault A Thuong M . Prélèvement d’organes en France: état des lieux et perspectives. La Presse Médicale (2013) 42(3):295–308. 10.1016/j.lpm.2012.05.018 22824722

[9] Hoogland ERP Snoeijs MGJ Winkens B Christaans MHL Van Heurn LWE . Kidney transplantation from donors after cardiac death: uncontrolled *versus* controlled donation. Am J Transplant (2011) 11(7):1427–34. 10.1111/j.1600-6143.2011.03562.x 21668628

[10] Summers DM Watson CJE Pettigrew GJ Johnson RJ Collett D Neuberger JM Kidney donation after circulatory death (DCD): state of the art. Kidney Int (2015) 88(2):241–9. 10.1038/ki.2015.88 25786101

[11] Moers C Smits JM Maathuis MHJ Treckmann J Van Gelder F Napieralski BP Machine perfusion or cold storage in deceased-donor kidney transplantation. N Engl J Med (2009) 360(1):7–19. 10.1056/NEJMoa0802289 19118301

[12] Phillips BL Ibrahim M Greenhall GHB Mumford L Dorling A Callaghan CJ . Effect of delayed graft function on longer-term outcomes after kidney transplantation from donation after circulatory death donors in the United Kingdom: a national cohort study. Am J Transplant (2021) 21(10):3346–55. 10.1111/ajt.16574 33756062

[13] Cantafio AW Dick AAS Halldorson JB Bakthavatsalam R Reyes JD Perkins JD . Risk stratification of kidneys from donation after cardiac death donors and the utility of machine perfusion. Clin Transplant (2011) 25(5):E530–E540. 10.1111/j.1399-0012.2011.01477.x 21585547

[14] Locke JE Segev DL Warren DS Dominici F Simpkins CE Montgomery RA . Outcomes of kidneys from donors after cardiac death: implications for allocation and preservation. Am J Transplant (2007) 7(7):1797–807. 10.1111/j.1600-6143.2007.01852.x 17524076

[15] Aiello S Cassis P Mister M Solini S Rocchetta F Abbate M Rabbit anti-rat thymocyte immunoglobulin preserves renal function during ischemia/reperfusion injury in rat kidney transplantation: thymoglobuline in post-transplant I/R injury. Transpl Int (2011) 24(8):829–38. 10.1111/j.1432-2277.2011.01263.x 21545548

[16] Sabah TK Khalid U Ilham MA Ablorsu E Szabo L Griffin S Induction with ATG in DCD kidney transplantation; efficacy and relation of dose and cell markers on delayed graft function and renal function. Transpl Immunol (2021) 66:101388. 10.1016/j.trim.2021.101388 33775865

[17] Hullegie-Peelen DM Hesselink DA Dieterich M Minnee RC Peeters A Hoogduijn MJ Tissue-resident lymphocytes are released during hypothermic and normothermic machine perfusion of human donor kidneys. Transplantation (2024). 108(7). 10.1097/TP.0000000000004936 38557650 PMC11188625

[18] Larsen CP Morris PJ Austyn JM . Migration of dendritic leukocytes from cardiac allografts into host spleens. A novel pathway for initiation of rejection. The J Experimental Medicine (1990) 171(1):307–14. 10.1084/jem.171.1.307 2404081 PMC2187651

[19] Charmetant X Chen CC Hamada S Goncalves D Saison C Rabeyrin M Inverted direct allorecognition triggers early donor-specific antibody responses after transplantation. Sci Transl Med (2022) 14(663):eabg1046. 10.1126/scitranslmed.abg1046 36130013

[20] Charmetant X Pettigrew GJ Thaunat O . Allorecognition unveiled: integrating recent breakthroughs into the current paradigm. Transpl Int (2024) 37:13523. 10.3389/ti.2024.13523 39588197 PMC11586167

[21] Naesens M Roufosse C Haas M Lefaucheur C Mannon RB Adam BA The banff 2022 kidney meeting report: reappraisal of microvascular inflammation and the role of biopsy-based transcript diagnostics. Am J Transplant (2024) 24(3):338–49. 10.1016/j.ajt.2023.10.016 38032300

[22] R Core Team. R: A Language and Environment for Statistical Computing. Vienna: R Foundation for Statistical Computing (2023). Available online at: https://www.R-project.org/ (Accesssed February 2, 2026).

[23] Protocole des conditions à respecter pour réaliser des. Agence de la biomédecine (2003). Available online at: https://www.agence-biomedecine.fr/Protocole-des-conditions-a-respecter-pour-realiser-des-prelevements-d-organes (Accessed April 14, 2025).

[24] Thaunat O Legeai C Anglicheau D Couzi L Blancho G Hazzan M IMPact of the COVID-19 epidemic on the moRTAlity of kidney transplant recipients and candidates in a French nationwide registry sTudy (IMPORTANT). Kidney Int (2020) 98(6):1568–77. 10.1016/j.kint.2020.10.008 33137341 PMC7604114

[25] Caillard S Anglicheau D Matignon M Durrbach A Greze C Frimat L An initial report from the French SOT COVID registry suggests high mortality due to COVID-19 in recipients of kidney transplants. Kidney Int (2020) 98(6):1549–58. 10.1016/j.kint.2020.08.005 32853631 PMC7444636

[26] Brennan DC Daller JA Lake KD Cibrik D Del Castillo D , Thymoglobulin Induction Study Group. Rabbit antithymocyte globulin *versus* basiliximab in renal transplantation. N Engl J Med (2006) 355(19):1967–77. 10.1056/NEJMoa060068 17093248

[27] Fuquay R Renner B Kulik L McCullough JW Amura C Strassheim D Renal ischemia-reperfusion injury amplifies the humoral immune response. J Am Soc Nephrol (2013) 24(7):1063–72. 10.1681/ASN.2012060560 23641055 PMC3699821

[28] Andrews PA Burnapp L Manas D British Transplantation Society. Summary of the British transplantation society guidelines for transplantation from donors after deceased circulatory death. Transplantation (2014) 97(3):265–70. 10.1097/01.TP.0000438630.13967.c0 24448588

[29] Van Heurn LWE Talbot D Nicholson ML Akhtar MZ Sanchez-Fructuoso AI Weekers L Recommendations for donation after circulatory death kidney transplantation in Europe. Transpl Int (2016) 29(7):780–9. 10.1111/tri.12682 26340168

[30] Favi E Puliatti C Iesari S Monaco A Ferraresso M Cacciola R . Impact of donor age on clinical outcomes of primary single kidney transplantation from maastricht Category-III donors after circulatory death. Transplant Direct (2018) 4(10):e396. 10.1097/TXD.0000000000000835 30498772 PMC6233668

[31] Asderakis A Sabah TK Watkins WJ Khalid U Szabo L Stephens MR Thymoglobulin *versus* alemtuzumab *versus* basiliximab kidney transplantation from donors after circulatory death. Kidney Int Rep (2022) 7(4):732–40. 10.1016/j.ekir.2022.01.1042 35497810 PMC9039467

[32] Oniscu GC Randle LV Muiesan P Butler AJ Currie IS Perera MTPR *In situ* normothermic regional perfusion for controlled donation after circulatory death—the United Kingdom experience. Am J Transplant (2014) 14(12):2846–54. 10.1111/ajt.12927 25283987

[33] Thaunat O Legendre C Morelon E Kreis H Mamzer-Bruneel MF . To biopsy or not to biopsy? Should we screen the histology of stable renal grafts? Transplantation (2007) 84(6):671–6. 10.1097/01.tp.0000282870.71282.ed 17893596

[34] Michallet MC Preville X Flacher M Fournel S Genestier L Revillard JP . Functional antibodies to leukocyte adhesion molecules in antithymocyte globulins1. Transplantation (2003) 75(5):657–62. 10.1097/01.TP.0000053198.99206.E6 12640305

[35] Bonnefoy-Bérard N Vincent C Revillard JP . Antibodies against functional leukocyte surface molecules in polyclonal antilymphocyte and antithymocyte globulins. Transplantation (1991) 51(3):669–73. 10.1097/00007890-199103000-00024 2006524

[36] Lebranchu Y Bridoux F Büchler M Meur YL Etienne I Toupance O Immunoprophylaxis with basiliximab compared with antithymocyte globulin in renal transplant patients receiving MMF-Containing triple therapy. Am J Transplant (2002) 2(1):48–56. 10.1034/j.1600-6143.2002.020109.x 12095056

[37] Mourad G Rostaing L Legendre C Garrigue V Thervet E Durand D . Sequential protocols using basiliximab *versus* anti-thymocyte globulins in renal-transplant patients receiving mycophenolate mofetil and steroids. Transplantation (2004) 78(4):584–90. 10.1097/01.tp.0000129812.68794.cc 15446319

[38] Ciancio G Burke GW Gaynor JJ Carreno MR Cirocco RE Mathew JM A randomized trial of three renal transplant induction antibodies: early comparison of tacrolimus, mycophenolate mofetil, and steroid dosing, and newer Immune-Monitoring1. Transplantation (2005) 80(4):457–65. 10.1097/01.tp.0000165847.05787.08 16123718

[39] Mourad G Garrigue V Squifflet JP Besse T Berthoux F Alamartine E Induction *versus* noninduction in renal transplant recipients with tacrolimus-based immunosuppression1. Transplantation (2001) 72(6):1050–5. 10.1097/00007890-200109270-00012 11579299

[40] Charpentier B Rostaing L Berthoux F Lang P Civati G Touraine JL A three-arm study comparing immediate tacrolimus therapy with antithymocyte globulin induction therapy followed by tacrolimus or cyclosporine A in adult renal transplant recipients1. Transplantation (2003) 75(6):844–51. 10.1097/01.TP.0000056635.59888.EF 12660513

[41] Bamoulid J Staeck O Crépin T Halleck F Saas P Brakemeier S Anti-thymocyte globulins in kidney transplantation: focus on current indications and long-term immunological side effects. Nephrol Dial Transpl (2016) 32(10):1601–8. 10.1093/ndt/gfw368 27798202

[42] Thangaraju S Gill J Wright A Dong J Rose C Gill J . Risk factors for BK polyoma virus treatment and association of treatment with kidney transplant failure: insights from a paired kidney analysis. Transplantation (2016) 100(4):854–61. 10.1097/TP.0000000000000890 27003098

[43] Schold JD Rehman S Kayler LK Magliocca J Srinivas TR Meier-Kriesche HU Treatment for BK virus: incidence, risk factors and outcomes for kidney transplant recipients in the United States. Transpl Int (2009) 22(6):626–34. 10.1111/j.1432-2277.2009.00842.x 19207187

[44] Prince O Savic S Dickenmann M Steiger J Bubendorf L Mihatsch MJ . Risk factors for polyoma virus nephropathy. Nephrol Dial Transplant (2009) 24(3):1024–33. 10.1093/ndt/gfn671 19073658 PMC2644630

